# Supplementary material for: Conspiracy theories as engines of connection for enriched public debates on emerging technologies
Source: Commun Earth Environ. 2025 Aug 13;6(1):655. doi: 10.1038/s43247-025-02581-x (PMC12350156; doi:10.1038/s43247-025-02581-x)
Supplement: Supplementary file 2 — Supplementary material [file 43247_2025_2581_MOESM2_ESM.docx]

Gabriel Dorthe
ETH Zürich, D-GESS (Department of Humanities, Social and Political Sciences)
Zürich, Switzerland
gabriel.dorthe@gmail.com

**Conspiracy theories as engines of connection for enriched public debates on emerging technologies**

**– Supplementary Materials –**

**List of blogs, social media pages and groups (all URLs verified on December 6, 2024):**

**Chemtrails and solar geoengineering:**

Blogs:

French association Ciel Voilé: www.cielvoile.fr

US-based association Geoengineering Watch: www.geoengineeringwatch.org

French Association Citoyenne pour le Suivi, L’Étude et l’Information sur les Programmes d’Interventions Climatiques et Atmosphériques: www.acseipica.fr

Tanker Enemy: http://tankerenemy.blogspot.com

Climate Change Agenda: https://climatechangeagenda.com

UK-based organization: www.look-up.org.uk

Social media pages and groups:

Facebook group *Geoengineering Global Skywatch*: www.facebook.com/groups/293625607452697

Facebook group *don't touch my Sky !*: www.facebook.com/groups/donttouchmysky/about

Facebook group *Chemtrail Cures*: www.facebook.com/groups/chemtrailcures/about

Facebook group *Chemtrails Benelux*: www.facebook.com/groups/195559833907253/about

Facebook group *Chemtrails Schweiz*: www.facebook.com/groups/547176788692635/about

Facebook page *Global MARCH Against Chemtrails and Geoengineering – Berlin*: www.facebook.com/marchagainstchemtrailsberlin/about

Facebook group *Mass Action Demand: Chemtrails & Geoengineering & Democracy*: www.facebook.com/groups/ChemtrailHypernetwork/about

Facebook group *CHEMTRAILS INTERNATIONAL*: www.facebook.com/groups/CHEMTRAILSINTERNATIONAL/about

Facebook group *ACSEIPICA*: www.facebook.com/groups/Acseipica.Association

Telegram channel *Chem.trails/HAARP-Forschung*

**COVID vaccines:**

Blogs:

Who is Robert Malone: www.malone.news

Dr. Mercola: www.mercola.com

Natural Blaze: https://naturalblaze.com

Project Veritas: www.projectveritas.com

The Epoch Times United States edition: www.theepochtimes.com

Social media pages and groups:

Facebook group *Les NoN-Vaccinés*: www.facebook.com/groups/2974266466119610

Facebook page *Pierre Barnérias*: www.facebook.com/tprodofficiel

Telegram channel *Dr. Carrie Madej*: https://t.me/carriemadej

Telegram channel *Témoignages vax-shedding francophones*: https://t.me/temoignageshedding

Telegram channel *Live a healthy life*: https://t.me/Live_a_healthy_life1

Telegram channel *The Great Awakening Global*: https://t.me/TheGreatAwakeningGlobal

Telegram channel *Project Veritas*: https://t.me/project_veritas

Telegram channel *BonSens.org Officiel*: https://t.me/BonSens_org

Telegram channel *Free Radio Truth Stream*: https://t.me/freeradiotruthstream

Telegram channel *COVID-19 Up*: https://t.me/COVID19Up

Telegram group *COVID-19 Chat*: https://t.me/COVID1984chat
